# The peritoneal macrophage inflammatory profile in cirrhosis depends on the alcoholic or hepatitis C viral etiology and is related to ERK phosphorylation

**DOI:** 10.1186/1471-2172-13-42

**Published:** 2012-08-06

**Authors:** Ana Tapia-Abellán, María Martínez-Esparza, Antonio J Ruiz-Alcaraz, Trinidad Hernández-Caselles, Cristina Martínez-Pascual, Manuel Miras-López, José Such, Rubén Francés, Pilar García-Peñarrubia

**Affiliations:** 1 Departamento de Bioquímica, Biología Molecular (B) e Inmunología Facultad de Medicina, Universidad de Murcia, Murcia, 30100, Spain; 2 Unidad de Trasplante Hepático, Servicio de Aparato Digestivo, Hospital Universitario Virgen de la Arrixaca, Murcia, Spain; 3 Unidad Hepática, Hospital General Universitario, Alicante, Spain; 4 CIBERehd, Instituto de Salud Carlos III, Madrid, Spain

**Keywords:** Ascites, Cirrhosis, Cytokines, Etiology, MAP kinases, HCV, Alcohol

## Abstract

**Background:**

The development of ascites in cirrhotic patients generally heralds a deterioration in their clinical status. A differential gene expression profile between alcohol- and hepatitis C virus (HCV)-related cirrhosis has been described from liver biopsies, especially those associated with innate immune responses. The aim of this work was to identify functional differences in the inflammatory profile of monocyte-derived macrophages from ascites in cirrhotic patients of different etiologies in an attempt to extrapolate studies from liver biopsies to immune cells in ascites. To this end 45 patients with cirrhosis and non-infected ascites, distributed according to disease etiology, HCV (n = 15) or alcohol (n = 30) were studied. Cytokines and the cell content in ascites were assessed by ELISA and flow cytometry, respectively. Cytokines and ERK phosphorylation in peritoneal monocyte-derived macrophages isolated and stimulated *in vitro* were also determined.

**Results:**

A different pattern of leukocyte migration to the peritoneal cavity and differences in the primed status of macrophages in cirrhosis were observed depending on the viral or alcoholic etiology. Whereas no differences in peripheral blood cell subpopulations could be observed, T lymphocyte, monocyte and polymorphonuclear cell populations in ascites were more abundant in the HCV than the alcohol etiology. HCV-related cirrhosis etiology was associated with a decreased inflammatory profile in ascites compared with the alcoholic etiology**.** Higher levels of IL-10 and lower levels of IL-6 and IL-12 were observed in ascitic fluid from the HCV group. Isolated peritoneal monocyte-derived macrophages maintained their primed status *in vitro* throughout the 24 h culture period. The level of ERK1/2 phosphorylation was higher in ALC peritoneal macrophages at baseline than in HCV patients, although the addition of LPS induced a greater increase in ERK1/2 phosphorylation in HCV than in ALC patients.

**Conclusions:**

The macrophage inflammatory status is higher in ascites of alcohol-related cirrhotic patients than in HCV-related patients, which could be related with differences in bacterial translocation episodes or regulatory T cell populations. These findings should contribute to identifying potential prognostic and/or therapeutic targets for chronic liver diseases of different etiology.

## Background

Alcoholic liver disease (ALD) and hepatitis C virus (HCV) infection are the two major causes of chronic liver diseases in the developed world [1]. The development of ascites, an accumulation of fluid in the peritoneal cavity, in a cirrhotic patient generally runs parallel with deterioration in clinical status and presages a poor prognosis. While the overall cirrhosis mortality rate has falling in the last three decades [2], HCV mortality rates, closely associated with cirrhosis, have been increasing since the 1990s [3]. For the clinical management of cirrhosis, it is important to assess whether the differences in the outcomes of cirrhosis depend on the etiology. In this respect, the histopathology of alcohol-induced cirrhosis (ALC-C) and HCV-induced cirrhosis (HCV-C) is very similar and characteristic fibrosis patterns leading to cirrhosis can overlap in them [4]. Nonetheless, little is known regarding the differences and/or similarities between alcohol- and HCV- induced liver disease at the molecular level [5] and, to the best of our knowledge, no data on human peritoneal cells have bee reported.

Global transcriptional profiling using oligonucleotide microarrays on liver biopsies from patients with cirrhosis showed that some genes are differentially expressed between ALC-C and HCV-C. Many of the gene expression changes specifically observed in HCV-induced cirrhotic livers were related to activation of the innate antiviral immune response, while differential mechanisms between chronic liver damage due to HCV or ethanol may be related to regulation of the lipid metabolism and macrophage activation resulting in the deposition of extracellular matrix components [1,5]. Additionally, several studies have demonstrated that alcohol exposure activates innate immunity and induces several pro-inflammatory cytokines, including TNF-α, subsequently inducing hepatocellular damage. The activation of innate immunity also results in higher levels of hepatoprotective intermediaries, such as IL-6, and anti-inflammatory cytokines, such as IL-10, which play an important role in ameliorating alcoholic liver injury and inflammation [6]. However, chronic alcohol exposure attenuates the signaling pathways triggered by these cytokines, thereby limiting their anti-inflammatory and hepatoprotective effects, and contributing to the development of ALD [7].

Monocyte-derived macrophages (M-DM) play an important role as antigen-presenting cells of the innate immune response established against pathogen associated molecular patterns (PAMPs), including bactDNA and LPS [8]. In this respect, we reported bactDNA-activated cell-mediated immune response and nitric oxide overproduction through the inducible form of NO synthase in peritoneal macrophages from patients with cirrhosis and ascites [9] and, more recently, the primed status of peritoneal M-DM from cirrhotic patients related to ERK phosphorylation and IL-6 secretion [10].

This study focuses on the inflammatory profile of ascites and peritoneal M-DM from patients with ALC-C or HCV-C to assess whether the immune status and inflammatory mechanisms leading to end-stage liver cirrhosis present a different pattern, depending on the agent that caused this pathology, and extrapolate out from previous studies performed in liver biopsies to immune cells in ascites.

## Results

### Patient baseline characteristics

A consecutive series of 61 patients with cirrhosis and ascites was initially recruited for the study. Sixteen patients were excluded due to episodes of gastrointestinal bleeding in the previous 2 weeks (n = 2), culture-positive ascites (n = 3), hepatocellular carcinoma (n = 2), mixed alcoholic and viral etiology (n = 4), hepatitis B virus infection etiology (n = 1) or continuous use of norfloxacin as secondary prophylaxis of spontaneous bacterial peritonitis (n = 4). Finally 45 patients fulfilling all the inclusion criteria were included in the study, distributed according to disease etiology, HCV-C (n = 15) or ALC-C (n = 30). The clinical and analytical characteristics of patients are detailed in Table 1. Blood and ascites cultures were negative in all cases. None of the patients died during hospitalization or developed spontaneous bacterial peritonitis.

**Table 1:** Clinical and analytical characteristics of patients included in the study

**Variable**	**Patients with cirrhosis and culture-negative ascites (n = 45)**	
**Etiology**	**ALC-C (30)**	**HCV-C (15)**
Age	58.5 (12.5)	58 (24)
Male sex n (%)	30 (100.0)	14 (93.3)
Previous episodes of ascites n (%)	25 (83.3)	13 (86.6)
Child-Pugh mean score	10 (3)	9 (3)
Meld mean score	14.5 (10)	14 (5)
Bilirubin (mg/dl)	2.7 (3)	1.85 (1.7)
Albumin (g/dl)	2.9 (1.1)	2.85 (0.9)
Quick (%)	64 (18)	54 (24)
Serum creatinine (mg/dl)	1.07 (1.2)	1 (0.5)
Serum sodium (mEq/l)	134 (6.3)	136 (7)
INR	1.45 (0.3)	1.46 (0.6)
Blood WBC/mm^3^	4260 (2400)	3930 (4040)
Ascites WBC/mm^3^	21.29 (47.4)	49 (82.8)*
Ascites Total protein (g/dl)	1.7 (0.3)	1.6 (1)

Continuous variables are expressed as median (interquartile range) and categorical variables as percentage. WBC: white blood cells; Mann–Whitney U test * P < 0.05.

As can be observed from Table 1, the absolute total number of ascitic leukocytes found in patients with HCV-C was significantly higher than in ALC-C patients. These differences were not detected in blood.

There were no significant differences as regards the rest of the clinical and analytical characteristics between the two groups of patients studied.

During 6-month follow-up study, three patients from the ALC-C group and one patient from the HCV-C group died. The causes of death in the ALC-C group were liver insufficiency (n = 2) and renal failure (n = 1). In the HCV-C group, the cause of death was renal failure (n = 1).

### HCV-C is associated with increased counts of some leukocyte subpopulations in ascites

To further explore differences in the number of leukocytes present in the ascites of both groups, the leukocyte subpopulations were characterized by flow cytometry analysis based on both the morphology and CD receptor expression criteria. The results presented in Table 2 show that T lymphocytes, PMNs and M-DM counts were high in the ascites of HCV-C patients compared with the ALC-C group. A study of subset cell type distribution in blood showed no significant differences between ALC-C and HCV-C patients (data not shown).

**Table 2:** White blood cell distribution in ascites

	**Etiology**	
**Cell type/mm**^**3**^	**ALC-C**	**HCV-C**
PMNs	0.5 (4.4)	3.4 (7)*
M-DM	6.95 (12.6)	14.8 (19.9)*
T Lymphocytes	7.37 (12.6)	14,66 (22.6)*
B Lymphocytes	0.16 (0.5)	0.21 (0.3)
NK cells	1.22 (2.2)	2.01 (5.6)

Results are expressed as median (interquartile range). Mann–Whitney U test: * P < 0.05.

### Anti-inflammatory cytokine levels are higher and pro-inflammatory levels are lower in the ascites of HCV-C cirrhotic patients

**Figure 1:**
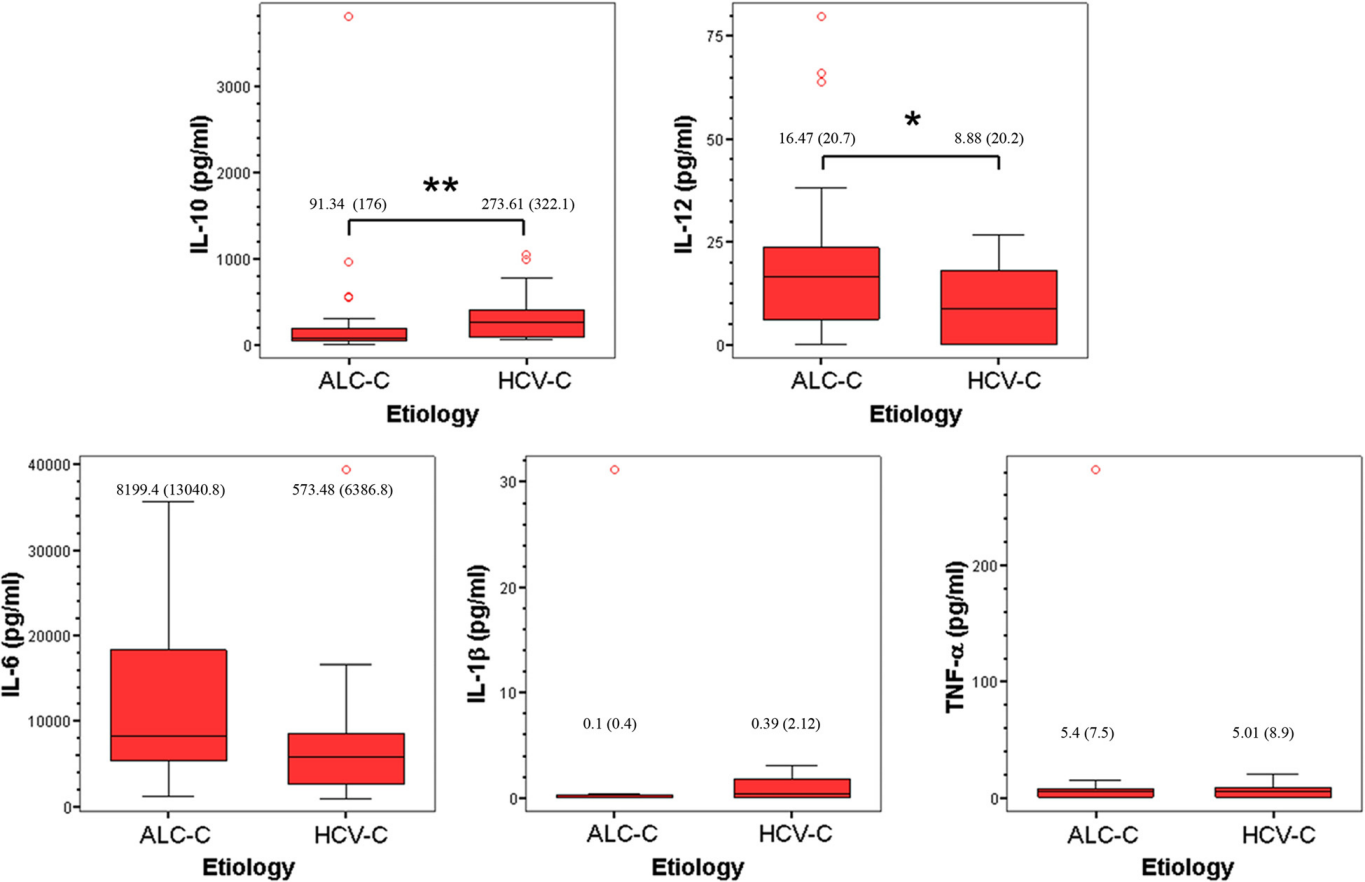
**Levels of pro- and anti-inflammatory cytokines in ascites from patients with HCV-C or ALC-C.** Cytokine concentration was measured by ELISA in ascites from patients with HCV-C (n = 15) or ALC-C (n = 30). Results are expressed in pg/ml and represented as box plot. Circles out of the boxes correspond to outliers. The median and IQR are indicated for each box. Mann–Whitney *U* test: * P < 0.05, ** P < 0.01.

Figure 1 shows the concentration in pg/ml of IL-10, IL-12, IL-1β, IL-6 and TNF-α. The results showed that the ascites from the HCV-C group had lower levels of pro-inflammatory cytokines, although the differences between both groups were statistically significant only in the case of IL-12. The results also revealed that IL-10 anti-inflammatory cytokine levels in the ascites of HCV-C patients were significantly higher than the corresponding levels from the ALC-C group of cirrhotic patients.

### Peritoneal M-DM isolated from ascites of cirrhotic patients maintain their cytokine secretory profile *in vitro*

**Figure 2:**
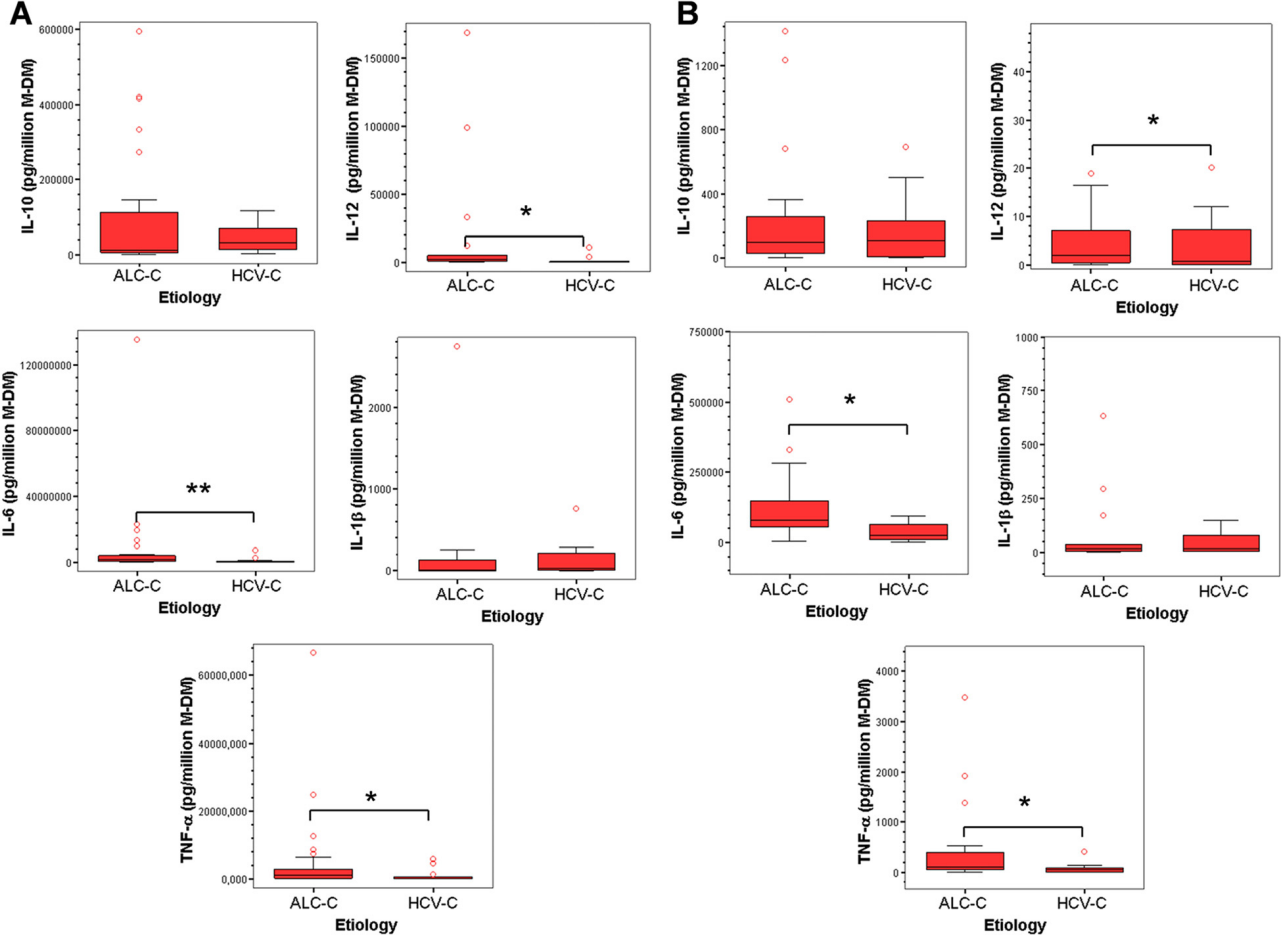
**Cytokine levels produced by Monocyte-Derived Macrophages from ascites of patients with HCV-C or ALC-C. A)** Cytokine concentration was measured by ELISA in ascites from patients with HCV-C (n = 15) or ALC-C (n = 30) and results were related to M-DM present in ascites. **B)** M-DM present in ascites were isolated as indicated in Methods and the cytokine concentration was measured by ELISA in cell culture supernatants after 24 h. Results are expressed in pg/10^6^ M-DM and represented as box plot. Circles out of the boxes correspond with outliers. Mann–Whitney *U* test:* P < 0.05, ** P < 0.01.

We next compared the cytokine production ability of the peritoneal M-DM from HCV-C and ALC-C patients, first exploring the relative contribution of the M-DM to the cytokines contained in ascites by referring the concentration of cytokines to the number of this cell type (Figure 2A). Then we analyzed the basal production of cytokines in 24 h cultures of the M-DM population isolated from ascites (Figure 2B).

The results revealed that M-DM from the HCV-C group produces significantly lower levels of IL-12, but also of IL-6 and TNF-α cytokines, than the ALC-C group when M-DM were isolated and cultured *in vitro* (Figure 2B). These data are consistent with those of cytokine levels in the ascites (Figure 2A), indicating that M-DM are “primed” by the pathological microenvironment and preserve their secretory profile, being more pro-inflammatory in the case of ALC-C group.

No significant differences in IL-10 production were observed between the two groups as regards a M-DM cultured *in vitro*, nor with respect to M-DM present in ascites, indicating that M-DM are not the main cell type responsible for the differences found for this cytokine in ascites.

### Peritoneal M-DM isolated from ascites of cirrhotic patients are able to further respond to *in vitro* stimulation with several microbial stimuli

Spontaneous bacterial peritonitis (SBP) caused by the translocation of intestinal bacteria and their products, such as LPS or bacterial DNA, is one of the main life-threatening complications of cirrhosis. Given this, the quality and intensity of the peritoneal immune response plays a crucial role in the prognosis of these compromised patients. To seek further insight into the ability of cirrhotic peritoneal M-DM to respond to different PAMPs, we studied the capability of isolated M-DM to respond to LPS, synthetic CpG-ODNs and heat-killed *C. albicans* as examples of stimuli able to trigger activation and phagocytosis through different PRRs. For this purpose, the cytokines produced by M-DM cultured for 24 h in the presence of these stimuli were studied. The results shown in Table 3 reveal that this cell population is able to further recognize and respond to different PAMPs, although significant differences were observed, depending on the cytokine in question and the specific stimuli applied. The results shown in Table 3 point to a, first strong significant response of TNF-α secretion in the presence of LPS and *C. albicans* in M-DM from both groups of patients. Second, IL-6 and IL-10 secretion was significantly increased in all stimulatory conditions, except in the M-DM from ALC-C patients after stimulation by ODN. Third, the relative increases in IL-6 was significantly higher in HCV-C for the three stimuli assayed, while the increase in IL-10 secretion was only significant in the ALC-C group stimulated by ODN. Fourth, secreted IL-12 was only significantly increased by LPS and *C. albicans* in M-DM obtained from ALC-C patients. Finally, LPS and *C. albicans* induced a significant increase in secreted IL-1β in M-DM from both groups of patients compared with their respective controls, although no significant differences were observed between groups.

**Table 3:** **Monocyte-Derived Macrophages response t o*in vitro *stimulation**

**Cytokines**	**Etiology**		**Fold activation**	
		**LPS**	**ODN**	***C. albicans***
**TNF-α**	ALC-C	89.26 (204.5) ^**a**^	0.89 (1.7)	170.92 (374.6) ^**a**^
	HCV-C	97.61 (163.9) ^**a**^	2.42 (4.9)	4334.96 (595.4) ^**a**^
**IL-6**	ALC-C	2.73 (3.5) ^**a**^	0.84 (5.8)	3.35 (10.4) ^**a**^
	HCV-C	5.97 (19.6) *^,a^	1.26 (7.2)*^,a^	5.68 (37.2) *^,a^
**IL-10**	ALC-C	9.06 (6.2)^**a**^	0.77 (0.6)	13.7 (24.5) ^**a**^
	HCV-C	11.33 (13.9) ^**a**^	1.4 (2.6) *^,a^	223.4 (45.3) ^**a**^
**IL-12**	ALC-C	1.75 (3.4) ^**a**^	2.1 (8.4)	3.7 (6.6) ^a,^*
	HCV-C	3.15 (4.8)	6.6 (17.4)	1.93 (1.1)
**IL-1β**	ALC-C	20.21 (63) ^**a**^	1.5 (10)	46.85 (135.6) ^**a**^
	HCV-C	10.89 (24.7) ^**a**^	2.11 (2.73)	15.4 (28.5) ^**a**^

Results are expressed as median (interquartile range). Mann–Whitney U test: * P < 0.05 between ALC-C and HCV-C; Wilcoxon signed-rank test:^*a*^*P <0.05 between control and treatments.*

### ERK1/2 phosphorylation levels are lower in peritoneal M-DM isolated from ascites of patients with HCV-C *vs.* ALC-C

**Figure 3:**
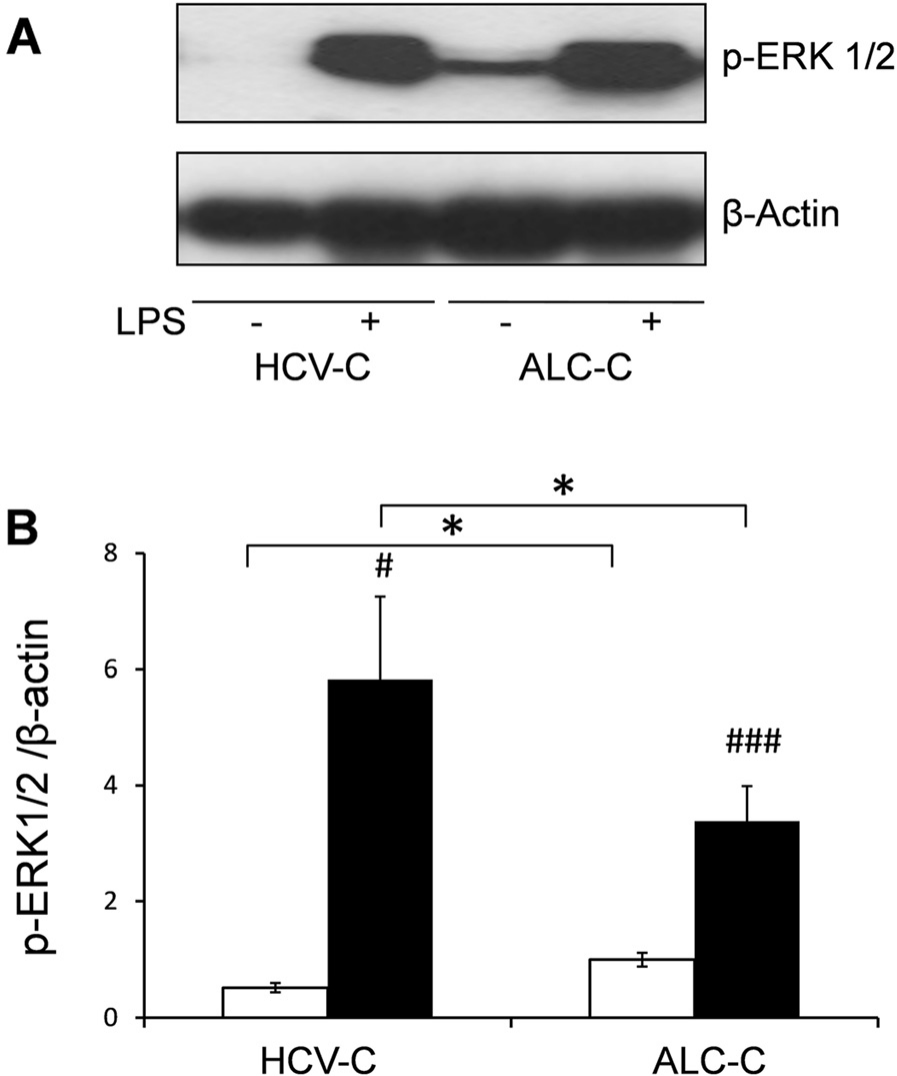
**ERK1/2 phosphorylation levels in Monocyte-Derived Macrophages from ascites of patients with HCV-C or ALC-C. A)** Representative immunoblots for p-ERK1/2 of peritoneal M-DM from patients with HCV-C (n = 4) or ALC-C (n = 12) at baseline and following LPS treatment. β-actin was measured as loading control. **B)** Immunoblots were quantified and the average ± SEM ratios of phosphorylation are represented as bar graphs referred to β-actin. Wilcoxon signed-rank test: # P < 0.05, ###P < 0.001, between control (normalized as 1) and treatments. Mann–Whitney *U* test: *P < 0.05, between ALC-C and HCV-C.

We recently described the primed status of peritoneal macrophages in cirrhosis, finding it to be related to ERK1/2 phosphorylation and IL-6 secretion [10]. We therefore further analyzed the phosphorylation of ERK1/2 at baseline and in the presence of LPS in M-DM from both group of patients. As shown in Figure 3, the results confirmed the primed status of ERK1/2 phosphorylation in peritoneal M-DM from ALC-C patients, which is consistent with the high concentration of IL-6 with respect to M-DM in ALC-C ascites, as well as with the IL-6 level produced *in vitro* by isolated M-DM cells from ALC-C patients. In contrast, baseline phosphorylation of ERK1/2 in M-DM from HVC-C patients was very low, although its relative increase after exposure to LPS for 15 min was higher than the corresponding increase in M-DM from ALC-C group. Again, this observation is consistent with the relative increase of IL-6 after LPS stimulation described above. These results confirm and extend the positive correlation previously established between basal IL-6 levels and ERK phosphorylation in peritoneal M-DM from patients with cirrhosis [10], and point to significant differences between ALC-C and HCV-C patients as regards the primed status at baseline.

## Discussion

Clinical research in human diseases is largely based on samples obtained by surgical or aggressive procedures from extremely ill patients. This implies the use of convenience samples of limited size rather than larger samples drawn at random from the population. Therefore, the main limitation of this type of study on the immune status in cirrhotic ascites is the nature and size of the sample. To minimize this limitation we processed consecutive samples obtained from cirrhotic patients from two different institutions during a three year period. A robust statistical test was used to ensure that the observed differences were significant. Nevertheless, this still does not solve the important question of whether results observed in samples of convenience can be generalized to the larger population. Herein we show first, that compared with ALC-C, HCV-C associated ascites contains a significantly higher number of leukocytes, although these findings did not match the cellular distributions observed in the peripheral blood. This indicates that the absolute cell number and population distribution of peritoneal leukocytes in cirrhotic ascites varies with the underlying cause and does not mirror the situation in peripheral blood. In this regard it is important to remark that the majority of studies on immune cells in human cirrhosis have been performed in blood samples, mainly due to the difficulty involved in obtaining and managing ascites [11,12] and in to a lesser extent, in liver biopsies [13,14]. Thus, it is interesting to further explore the ascites in order to extrapolate data on immune cells from the above three types of samples to better understand the mechanisms underlying the pathogenesis of liver damage, enabling doctors to predict the risks and outcomes of apply the appropriate treatments.

Furthermore the findings also reveal that leukocyte migration towards the peritoneal cavity is not a passive process induced by hemodynamic anomalies associated to cirrhosis, like portal hypertension, but more of an active chemoattractant-induced process, recruiting leukocytes not only from blood vessels, but also from impaired lymphatic drainage [15]. Moreover the higher number of leukocytes in ascites from HCV-C *vs.* ALC-C group resulted from increases in the T lymphocyte, PMN and monocyte cell subpopulations. This suggests that a specific differential pattern of chemoattractant stimuli must be involved in the recruitment of each particular cell population, depending on the microbial stimuli involved in the cirrhosis etiology. In this regard, it has been shown that the expression of CCR1, -2, -5 and CXC-1 is induced in patients with chronic liver diseases, so that intrahepatic increases of CCL3-5 and IL-8 chemokines would be involved in recruiting not only monocyte/macrophages but also other immune cell populations [16,17].

Our results also showed that ascites from HCV-C patients exhibits a significantly lower concentration of IL-12 pro-inflammatory cytokine and a significant higher amount of the anti-inflammatory cytokine IL-10 compared with the ALC-C group, suggesting a predominant Th2/Treg profile in the pathogenesis of advanced HCV-C. Indeed, it has been described that HCV by itself induces intrahepatic Tr1 cells [18], expands peripheral Treg in patients with normal aminotransferases [11] and affects dendritic cell function, switching the cytokine profile towards a suppressive phenotype of IL-10 and transforming growth factor beta predominance, preventing cell maturation and inhibiting the allostimulating capacity [19]. However, when cytokine levels were compared with the number of M-DM contained in ascites of these patients, or secreted *in vitro* by isolated peritoneal M-DM, apart from differences in IL-12, the concentration of IL-6 and TNF-α also significantly differed between both groups of patients. Furthermore, this fact was associated with lower baseline ERK1/2 phosphorylation than observed in the ALC-C group. Four conclusions can be drawn from these findings. First, these results confirm and extend the positive correlation established between basal IL-6 levels and ERK phosphorylation in peritoneal M-DM from patients with cirrhosis [10]. Second, they indicate that M-DM are “differently primed” by the *in vivo* pathophysiological environment and preserve their inflammatory differentiation profile for at least 24 h, being more pro-inflammatory in the case of ALC-C. This “alert state” could provide an advantage for preventing the development of SBP in intermittent events of intestinal bacterial translocation in ALC-C patients. Third, they confirm the predominantly immune inhibited status of M-DM in the end-stages of HCV-induced hepatic damage compared with ALC-C [20], which may be intended to prevent immune-mediated decompensation. Fourth, the drop of significant differences in IL-10 levels with respect to M-DM strongly suggests that other immune cells are contributing to the total amount of this anti-inflammatory cytokine in ascites of the HCV-C group, especially regulatory Th subpopulations. However, this does not mean that M-DM from HCV-C are functionally exhausted or endotoxin-tolerant as described [21], since they are able to further respond to stimulation by several PAMP agonists. In fact, the relative increase of IL-6 secretion and ERK1/2 phosphorylation in isolated M-DM stimulated by LPS was significantly higher in HCV-C than in ALC-C patients. The same results were obtained for IL-6 secretion induced by ODN and *C. albicans,* and IL-10 induced by ODN from the HCV-C group. The above findings also point to the differential expression and/or susceptibility to ligands of PRRs in the M-DM present in ascites from both types of cirrhotic patient. Supporting this hypothesis, the clear up-regulation of TLRs has been reported in specimens from patients with HCV infection [22,23] and ALC-induced liver damage (reviewed in [24]).

SBP caused by translocation of intestinal bacteria and their products such as LPS or bacterial DNA is one of the main life-threatening complications of cirrhosis [25]. Faced with this condition, the quality and intensity of the peritoneal immune response seemingly plays a crucial role in the prognosis of these compromised patients. The lower inflammatory profile of ascites of the HCV-C compared with alcoholic etiology could also be the result of the hypothetically lower frequency of intestinal bacterial translocation in HCV-C patients. Our results indicate the need for additional studies with a larger clinical sample to answer the question as to whether or not the difference in the MD-M peritoneal inflammatory status could influence the clinical outcome of patients beyond six months or the tolerance of a transplanted liver.

## Conclusions

This study shows for the first time, that the concentration of T lymphocyte, PMN and monocyte subpopulations in ascites of patients with HCV-C is higher than that of patients with ALC-C, while no significant differences exist in peripheral blood. This indicates the lack of correlation between circulating white blood cell numbers and peritoneal leukocytes in cirrhotic ascites. The study provides important information on some differential characteristics of cirrhotic peritoneal M-DM that will allow extrapolation to previous data obtained in peripheral blood and liver biopsies. This will contribute to a better understanding of the mechanisms underlying the pathogenesis of liver damage leading to decompensated cirrhosis of differing etiologies. Thus, HCV etiology is associated with a predominant immune inhibitory status in ascites and isolated peritoneal M-DM unlike the cirrhosis induced by alcohol. A better knowledge of the cellular and molecular pathways of M-DM activation in cirrhosis could contribute to the design of further studies to identify potential prognostic and/or therapeutic targets for liver fibrosis of different etiologies.

## Methods

### Patients

Patients were admitted at the Liver Unit of Hospital General Universitario, Alicante, Spain, or at the Liver Transplant Unit of Hospital Universitario Virgen de la Arrixaca, Murcia, Spain. Cirrhosis was diagnosed by histology or by clinical, laboratory, and/or ultrasonographic findings. Exclusion criteria were the presence of a culture-positive blood or ascites, an ascites polymorphonuclear (PMN) count equal or higher than 250/μl [26], signs or symptoms of systemic inflammatory response syndrome [27], upper gastrointestinal bleeding, hepatocellular carcinoma fulfilling Milan criteria [28] and/or portal thrombosis, previous liver transplantation, transjugular intrahepatic portosystemic shunt, alcoholic hepatitis, age older than 80 or younger than 18, etiology other than alcohol intake or HCV infection and refusal to participate in the study. The ethics committees (Comité Ético de Investigación Clínica del Hospital General de Alicante and Comité de Bioética de la Universidad de Murcia) approved the study protocol and all patients gave informed consent to be included in this study. A convenience sample of 61 consecutive patients who fulfilled all inclusion/exclusion criteria and who signed the informed consent were included in the study.

### Blood and ascites samples

Peripheral blood and ascites were collected from patients with cirrhosis requiring a large-volume paracentesis at admission.

Blood was obtained for routine haematological, biochemical and coagulation studies. Simultaneously, a large-volume paracentesis was performed on all patients at admission in aseptic conditions following the usual procedures to obtain ascites [29]. All patients received intravenous albumin after paracentesis (8 g/l of ascites) as routine protocol, if the volume of ascites evacuated was greater than 5 liters. Samples for routine biochemical study and PMN counts were obtained. Total protein, albumin, leukocyte and PMN counts were performed in all ascites specimens. Both blood and ascites were inoculated at bedside in aerobic and anaerobic blood culture bottles, 10 ml each [30].

### Flow cytometry analysis

Ascites samples were centrifuged at 500 × G, the supernatant was collected for cytokine detection and cells were collected and washed in PBS, and then resuspended in DMEM (GIBCO Invitrogen, Paisley, UK). Cells from ascites were stained with monoclonal antibodies and analyzed by flow cytometry to determine cell types. Antibodies were mouse anti-human CD14-FITC (eBioscience, San Diego, CA), CD3-FITC, CD19-PE Cy5, CD14-PE and CD16-PE Cy5 (BD-Pharmingen, San Diego, CA). The mouse IgG1-PE, mouse IgG1-FITC, mouse IgG1-PE Cy5, mouse IgG2b-FITC and mouse IgG2b-PE antibodies used as isotype controls were from BD-Pharmingen. In brief, 0.3 × 10^6^ cells in a volume of 100 μl were stained with 5 μl of the corresponding monoclonal antibodies and incubated in the dark on ice fixed in solution of lysis (Becton Dickinson, San José, CA) and then washed, resuspended in PBS and kept at 4°C in the dark until data acquisition.

Flow cytometry analyses were performed on three-color fluorescence Epics XL (Beckman Coulter) using Cytomics RXP analysis software. 50,000-100,000 gated events were acquired and analyzed. Leukocytes were gated based on FCS *vs.* SSC (Forward *vs.* Side Scatter) on a lineal scale. Then, leukocyte subpopulations were gated on the base of morphology and CD14^+^ expression for peritoneal M-DM, CD3^+^ expression for T lymphocytes, CD19^+^ expression for B lymphocytes and CD3^-^CD14^-^CD19^-^CD16^+^ expression for NK cells.

### Isolation and stimulation of M-DM

Cells were then seeded for panning at a ratio of 0.2 × 10^6^ M-DM/well in 96-well plates for ELISA, or 1-2 × 10^6^ M-DM/well in 6-well plates for immunoblotting, according to the percentage of CD14^+^ cells determined by flow cytometry. After an overnight incubation at 37°C in DMEM containing 10% fetal bovine serum and 1% penicillin/streptomycin (complete culture medium), cells were washed with complete culture medium to eliminate non-adhered cells, including lymphocytes. The purity of the M-DM in cell culture was more than 95%. Then M-DM were maintained for 3 hours in serum starvation conditions (2% FBS) and treated for 15 minutes with LPS (*E. coli* serotype 0111.B4, Sigma Aldrich Co, Saint Luis, Missouri, USA) for immunoblotting analysis of cell lysates, or directly with 0.1 μg/ml LPS, heat-killed *C. albicans* SC5314 strain at ratio 1/5 cell/yeasts and synthetic phosphorothioate oligodeoxynucleotides (ODN) (1 μg/ml) presenting the following sequence 5’ TGA CTG TGA ACG TTC GAG ATG A 3’ (TriLink BioTechnologies, San Diego, CA) for ELISA. After 24 hours of incubation, cell culture supernatants were collected for cytokine detection.

### ELISA

Ascitic fluid and cell culture supernatants were assayed by ELISA kits for TNF-α, IL-1β, IL-6, IL-10 and IL-12. The assay was performed in triplicate following the manufacturer’s instructions (©R&D Systems Inc., Minneapolis, USA). The absorbance in each well was measured with a microplate reader at 450 nm and corrected at 570 nm.

### Cellular lysates and immunoblotting

Protein extracts were obtained and treated as described elsewhere [31]. Primary antibodies against phosphorylated ERK1/2 (Thr202/Tyr204) and β-actin (Sigma Aldrich Co, Saint Luis, Missouri, USA) were used before incubation with the corresponding HRP-conjugated secondary antibody. The activity of membrane-bound peroxidase was detected by using an enhanced chemiluminescent detection method (Enhanced ChemiLuminescence system, ©Amersham Pharmacia Biotech; Piscataway, NJ, USA). Protein bands were quantified by densitometry using Scion Image software and expressed relative to β-actin.

### Statistical analysis

Categorical variables are reported as frequency or percentages. Continuous variables are reported as median and interquartile range (IQR) and are graphically represented as box diagrams, where the top and bottom of the box are the 25^th^ and 75^th^ percentile (the lower and upper quartiles, respectively), and the band inside the box is the 50^th^ percentile (the median), the end of the whiskers represent the lowest datum still within 1.5 IQR of the lower quartile, and the highest datum still within 1.5 IQR of the upper quartile. The observations considered as outliers are shown as circles outside the boxes. Statistic differences were analyzed using the Mann–Whitney *U* test or Wilcoxon signed-rank test. All reported P values are two-sided, and P values lower than 0.05 were considered to indicate statistical significance. All calculations were performed using the SPSS 15.0 software (Chicago, IL, USA).

Reporting of the study conforms to STROBE and EQUATOR guidelines [32].

## Abbreviations

ALD: Alcoholic liver disease; HCV: Hepatitis C Virus; ALC-C: Alcohol induced cirrhosis; HCV-C: HCV Induced Cirrhosis; M-DM: Monocyte-derived macrophages; PAMP: Pathogen-associated molecular pattern; PRR: PAMP-Recognition receptor; PMN: PolyMorphoNuclear; ODN: OligoDeoxyNucleotide; SBP: Spontaneous bacterial peritonitis.

## Competing interests

The authors declare that they have no competing interest.

## Authors’ contributions

ATA participated in the isolation and stimulation of M-DM, ELISA, immunoblotting and statistical analysis. MME participated in the isolation and stimulation of M-DM, ELISA, statistical analysis, the design of the study and drafted the manuscript, AJRA participated in the isolation and stimulation of M-DM and immunoblotting, THC carried out the flow cytometry analysis, CMP participated in the acquisition of blood and ascites samples, clinical and analytical data and informed consent from patients. MML participated in the design of the study. JS participated in the design of the study. RF participated in the acquisition of blood and ascites samples, clinical and analytical data, informed consent from patients and in the design of the study. PGP participated in the design and coordination of the study and helped to draft the manuscript. All authors read and approved the final manuscript.
